# Development of evidence-informed educational resources for advance care planning with older people with a mental illness

**DOI:** 10.1017/S1478951525100928

**Published:** 2025-10-23

**Authors:** Anne PF Wand, Roisin Browne, Yucheng Zeng, Aspasia Karageorge, Carmelle Peisah

**Affiliations:** 1 Specialty of Psychiatry, Faculty of Medicine and Health, University of Sydney CAR, Sydney, NSW, Australia; 2 School of Psychiatry and Mental Health, Faculty of Medicine and Health, University of New South Wales, Sydney, NSW, Australia; 3 Older Peoples Mental Health Service, Sydney Local Health District, Sydney, NSW, Australia; 4 Capacity Australia, Sydney, NSW, Australia; 5 Faculty of Medicine and Health, University of Sydney, Sydney, NSW, Australia; 6 Faculty of Medicine, Health and Human Sciences, Macquarie University, Sydney, NSW, Australia; 7 Sydney Brain and Mind Centre, The University of Sydney, Sydney, NSW, Australia

**Keywords:** Qualitative, death and dying, psychiatry, mental health, end-of-life

## Abstract

**Objectives:**

To triangulate the perspectives of mental health clinicians, older people with mental illness and their carers on Advance Care Planning (ACP) to develop evidence-informed educational resources.

**Methods:**

The study setting was public mental health services. Results of previously reported reflexive thematic analyses of interviews discussing ACP with three stakeholder groups (12 older people with mental illness, 5 carers, and 15 mental health clinicians) were triangulated. The emergent overarching themes were considered within an interpretive description framework to develop educational resources to support each of the three stakeholder groups to engage in ACP.

**Results:**

Four overarching themes emerged: (i) importance of ACP recognized but ACP often not initiated; (ii) knowledge gaps; (iii) skill gaps – how to do it; and (iv) practical and process issues. Taking into account the research team’s knowledge of the local health contexts, two formats of educational resources were developed; written information sheets bespoke to identified knowledge gaps and needs for each group, and brief training films for clinicians addressing need for practical skills in ACP. The consumer and carer sheets were translated into three languages. Two brief clinician training films demonstrated introducing ACP within mental health reviews and how to address aspects of complexity in ACP with older adults with a mental illness and carers.

**Significance of results:**

The current absence of specific educational resources for ACP with older people with mental illness contrasts with the recognized importance of ACP. Written resources were created to address empirically identified knowledge gaps and misconceptions and provide practical information and training films developed to demonstrate key skills for clinicians. The resources were made freely available, with dissemination planned to promote and evaluate use as part of a more comprehensive educational intervention. Resources supporting clinician, consumer and carer education are an important first step towards empowerment and participation in ACP.

## Introduction

The high prevalence of medical morbidity and premature mortality among people living with a mental illness (Firth et al. 2019) have created an imperative to improve quality care towards the end-of-life. Exercising autonomy in health-related decisions is one aspect of such care, acknowledged as a human right (Brennan 2007; Peisah et al. 2021). However, people with serious mental illness may not have their end-of-life needs recognized (Edwards et al. 2021) and are rarely engaged in ACP discussions (Appelbaum 2005; NSW Ministry of Health 2015a; Shalev et al. 2017).

Several studies have informed understanding of why ACP discussions do not occur with people with mental illness. Chief among the barriers are clinician assumptions about lack of decision-making capacity (Jerwood et al. 2018; Shalev et al. 2017; Wand et al. 2024, 2025), clinician (Jerwood et al. 2018; Wand et al. 2024, 2025; Woods et al. 2008) and carer (Wand et al. 2025) concerns about causing distress, deficiencies in clinician knowledge and training (Foti 2003; Wand et al. 2024; White et al. 2014) and mental health clinicians not considering ACP within their scope of practice (Edwards et al. 2021; McKellar et al. 2016; Wand et al. 2024). There is some overlap between mental health and general healthcare contexts regarding barriers to ACP implementation, such as lack of clinician knowledge and practical skills, uncertainty regarding clinician roles in ACP, questions about cognitive state and when to discuss ACP and insufficient time for ACP discussions (Poveda-Moral et al. 2021). However, in the general healthcare context, fears of depriving patients of hope and uncertainty about prognosis have been identified (Poveda-Moral et al. 2021). By contrast, mental health clinicians’ fears relate more to ACP discussions causing distress, making symptoms of mental illness worse and damaging the therapeutic relationship (Jerwood et al. 2018; Wand et al. 2024). Additionally, the complexity of assessing capacity in the context of persistent psychotic symptoms and cognitive impairment were emphasized by mental health clinicians (Wand et al. 2024). We have argued that many of these identified barriers reflect lack of knowledge about ACP with people with mental illness, where there are specific challenges and complexity, which are potentially remediable (Wand et al. 2024, 2025). Crucially, consumers and carers have indicated they consider mental health clinicians as best placed to help them with ACP (Wand et al. 2025), necessitating targeted training to prepare them for this role.

Despite the identified need to improve access to ACP for people with mental illness (Edwards et al. 2021), succinct and accessible evidence-based resources designed specifically to support mental health clinicians, consumers (patients) and carers to learn about and engage in ACP are scarce (Foti 2003). One exception is the “Do It Your Way” demonstration project which focused on end-of-life care for people with serious mental illness (Foti 2003). The project included establishing a coalition of interested parties, information meetings and education and training for providers of both mental health and palliative care/hospice services, with educational curricula for the provider groups (Foti 2003). Education included case studies and resource materials, including the “Do It Your Way” brochure and workbook for consumers, based on information requests and questions rather than empirical research (Foti 2003). Detailed content was not presented, and the resources are not freely accessible. Preliminary feedback on the curriculum was reportedly positive, although no quantitative results were published (Foti 2003). It is unclear whether the initial project – conducted over 20 years ago – was implemented into routine practice. More recently, New South Wales Health (Australia) published a comprehensive guide to ACP for end-of-life for people with mental illness for clinicians (46 pages) (NSW Ministry of Health 2015a) and an Introductory guide for consumers, families and carers (16 pages) (NSW Ministry of Health 2015b). These documents contain helpful information, but are lengthy, with no succinct “take-home” resources. Additionally, they utilize references and practical recommendations from the general literature, rather than grounding these in evidence specific to mental health.

The aim of this study was to triangulate the perspectives of mental health clinicians, older people with mental illness and their carers on Advance Care Planning to develop evidence-informed educational resources practically suitable for the local health context.

## Methods

This study describes the development of ACP educational resources informed by qualitative data from two previously reported empirical studies with multidisciplinary mental health clinicians (Wand et al. 2024) and older adult consumers of public mental health services and their carers (Wand et al. 2025). These previous studies explored perspectives on ACP with older people with a psychotic illness. The present study triangulates the themes which emerged in each of the three participant groups, exploring congruent and divergent themes. This novel analysis (triangulation) generated overarching themes, which were then applied to knowledge translation using an interpretive description framework to develop education and training resources. The original qualitative studies (Wand et al. 2024, 2025) and the present study were guided by the Consolidated Criteria for Reporting Qualitative Research (COREQ) (Tong et al. 2007) and were approved by the Human Research Ethics Committee – Concord Repatriation General Hospital of the Sydney Local Health District, Sydney, Australia (2023/ETH02283).

Three groups of participants were recruited from public mental health services across three Local Health Districts in Sydney, Australia. Participants were older adults with a psychotic illness (aged ≥55) [consumers] n = 12, their nominated adult carer n = 5, and multidisciplinary mental health clinicians n = 15. The characteristics of participants are summarized in Table 1. Each consumer and carer participated in an individual interview with an external clinician facilitator (Wand et al. 2025). Clinicians took part in focus groups with the same facilitator (Wand et al. 2024). The facilitator used a semi-structured interview guide to examine the experiences, attitudes, and perceived barriers and facilitators to ACP with participants. Individual interviews and focus group discussions were audio recorded and transcribed verbatim. Recruitment continued in all groups until saturation of themes was reached in data analysis.

**Table 1.** Characteristics of participants

Stakeholder group	Characteristics
Older people with a psychotic illness (consumers, n = 12)	Age: Mean 69 (range 60–75) Gender: 7 (58%) male, 5 (42%) female Marital status: 7 (58%) never married, 3 (25%) married, 2 (17%) divorced 8 different ethnicities 4 different religions Accommodation: 5 (42%) public housing, 4 (33%) private housing, 3 (25%) residential aged care facility Psychiatric diagnoses*: 6 (50%) schizophrenia, 3 (25%) depression with psychosis, 2 (17%) schizoaffective disorder, 2 (17%) very late onset schizophrenia like psychosis, 1 (8%) substance use disorder, 1 (8%) depression Medical comorbidity: mean 5.7 diagnoses/individual (range 0–11) Brief PANSS: mean 13.8 (range 7–27) CGI: mean 3.1 (range 0–5) Cognitive test score: mean MoCA 20.8/30 (range 13–27); 1 RUDAS 17/30
Nominated carers of older people with a mental illness (n = 5)	Gender: 4 (60%) male; 2 (40%) female 4 different ethnicities 4 different religions Relationship to consumer: 2 (40%) sibling, 2 (40%) paid carer, 1(20%) child 3 (60%) co-resided with the consumer
Mental health clinicians (n = 15)	Gender: 13 (87%) female, 2 (13%) male 6 different religions Mental health discipline: 5 (33%) social work, 4 (27%) psychiatrist, 3 (20%) nurse, 1(6%) clinical psychology, 1(6%) neuropsychology, 1(6%) occupational therapist 13 (87%) had prior training in ACP

Summarized from: (Wand et al. 2024, 2025).

* Some consumers had more than 1 psychiatric diagnosis.

Rating scales used: **PANSS** = Positive and Negative Symptom Scale (Yamamoto et al. 2010). The Brief PANSS is rated across 6 symptoms (e.g., unusual thought content, delusion), each scored from 1 (absent) to 7 (extreme). **CGI** = Clinical Global Impression Scale (Busner and Targum 2007). The CGI severity of illness scale is rated from 1 (normal) to 7 (among the most extremely mentally ill). A score of 0 indicates not assessed. **MoCA** = Montreal Cognitive Assessment (Nasreddine et al. 2005). A normal score is ≥26/30. **RUDAS** = Rowland Universal Dementia Assessment Scale (Storey et al. 2004). This test was used for participants whose first language was not English. A normal score is ≥26/30.

### Data analysis

Interview transcripts were analyzed using reflexive thematic analysis (Braun and Clarke 2006, 2019, 2021). Individual stakeholder group data (consumer, carer, clinician) have been previously reported (Wand et al. 2024, 2025). The qualitative analysis was grounded within an interpretive description framework; an approach which holds the creation of clinically- and practice-relevant knowledge central to the analysis (Thorne et al. 1997). This methodology is well suited to clinical translation by yielding practical applications relevant to service delivery and improvement. Two senior qualitative researchers (AW, CP) used constant comparison and regrouping of themes to derive triangulated themes in the present study.

#### Resource development

Triangulation refers to using multiple data sources or methods to develop in-depth understanding of phenomena (Carter et al. 2014). In this study, data source triangulation involved the analysis of data from the three stakeholder groups derived from focus groups (clinicians) (Wand et al. 2024) and individual interviews (consumers, carers) (Wand et al. 2025). Triangulation of qualitative data from the three groups led to identification of overarching themes relevant to implementation of ACP within clinical practice. Within an interpretive description framework for knowledge translation, this analysis was used to guide the development of resources to support implementation of ACP in a public mental health context. Each overarching theme was matched to an intervention to address the underlying issue (e.g., barriers or facilitators to ACP), taking into account practical considerations for the local mental health context.

## Results

Detailed results from the thematic analysis of the original qualitative studies have been reported (Wand et al. 2024, 2025). Key themes from the original studies of each group are summarized in Table 2.

**Table 2.** Emergent themes for consumers, carers and clinicians

Consumers (Wand et al. 2025) (i) What is ACP? (ii) I have not done ACP because…. Subthemes included (a) It’s not on my radar; (b) I didn’t know it was my choice; (c) Underestimation of the consumer (assumptions about mental illness and lack of capacity); (d) My General Practitioner is too busy; (e) It’s not time yet, I’m not dying; (f) ACP costs money (iii) I want to do ACP Subthemes included (a) It would be in my best interests; (b) Peace of mind for having my wishes respected; (c) Relieve family of burden (iv) If I was to do ACP I would need…. Subthemes included (a) It requires trust that it will be implemented; (b) I would want everyone involved; (c) Speaking to a professional about ACP; (d) Choosing where I do ACP; (e) Do it early; (f) Mental health clinicians have the skills to help me with ACP Carers (Wand et al. 2025) (i) We do not participate in ACP Subthemes included (a) Death and dying are too complex and confronting; (b) It might upset them; (c) Nobody brings it up; (d) General practitioners are too busy and unskilled; (e) ACP is only needed for people who are dying; (f) Not appropriate if cognitively impaired (ii) I want to participate in ACP Subthemes included (a) Families have important roles in ACP; (b) It will keep the consumer and me informed; (c) Empowerment and giving voice; (d) Peace of mind having wishes respected (iii) Key clinician skills are needed Subthemes included (a) Patience and attitude (recognizing mental health and cognitive symptoms fluctuate); (b) Cultural understanding and language; (c) Understanding trauma histories (prevalent in people with mental illness); (d) Flexible opportunities to do ACP Clinicians (Wand et al. 2024) (i) It is important, and I want to do it (ii) But I do not do it because of the complexity Subthemes in relation to this complexity included: (a) Fear of harming (causing distress/offence, exacerbating symptoms of mental illness, damaging the therapeutic relationship); (b) Families and culture; (c) Systemic barriers (ageism, discrimination on the basis of mental illness); (d) Capacity and legal issues (particularly in the context of persistent psychotic symptoms and/or cognitive impairment); (e) Timing of ACP (in relation to decision-making capacity and acuity of symptoms of mental illness); (f) Lack of knowledge and training; (g) Neither prioritized nor embedded in practice.

Triangulation of these themes from each of the stakeholder groups identified four overarching themes: (i) importance of ACP recognized but ACP often not initiated; (ii) knowledge gaps; (iii) skill gaps – how to do it; and (iv) practical and process issues. Each overarching theme is presented with illustrative quotes and discussed alongside a matched intervention to address the issue.

- Importance of ACP recognized but ACP not initiated

*“If you don’t have these conversations, the wishes of the patient can’t be fulfilled …. and the family need to know exactly where they’re coming from, what they want done.”* (female consumer, aged 67) (Wand et al. 2025)

*“[ACP] is sort of like giving the person a voice, a choice, while they’re still able to.”* (female, paid carer) (Wand et al. 2025)

*“For most people being able to have some autonomy or some ability to make decisions for their health is really important.”* (clinical psychologist) (Wand et al. 2024)

All stakeholder groups considered ACP important and empowering for the consumer. Consumers and carers highlighted ACP as relieving family of the burden of making such decisions, while clinicians identified that ACP may unearth family conflict and disagreement about end-of-life care.

Both consumers and carers identified that clinicians had not raised ACP with them. Inferences drawn from this included that it must not be relevant for the consumer (e.g., because their ability to engage in the process was underestimated and clinicians assumed mental illness equated to incapacity). Themes from clinician focus groups indicated that they thought ACP was relevant and important for their consumers, but complexity was a barrier to raising it.

### Matched intervention

These findings reflect the interest in and desire for ACP, with barriers for raising the issue with consumers. Interventions for this comprise training to address both knowledge and skill gaps which may be responsible for this reticence, as well as process issues such as ACP timing and incorporation into routine mental health reviews. These interventions are outlined in detail below.

- Knowledge gaps

All groups lacked knowledge about ACP and held various misconceptions such as consumers not having a choice in relation to end-of-life care, needing a lawyer, costs involved (consumers) and assumptions that cognitive impairment precluded engagement in ACP (carers). Clinicians shared some of these misconceptions (e.g., regarding consumer capacity to participate in ACP, especially when there are persistent psychotic symptoms), reflective of identified deficits in ACP education and training.

### Matched intervention

Key to this intervention was the development of free, easily accessible resources. To address the identified lack of ACP-related knowledge across all stakeholder groups a 2-page information sheet was devised, bespoke to each group. The intention was that clinicians could share these evidence-informed information sheets with consumers and carers, as a handout before or after ACP discussions to spark discussion and reinforce key information. The clinician information sheets were designed as a takeaway supplementary resource accompanying clinician education sessions. These bespoke handouts were tailored to the particular considerations for ACP with people with mental illness which drive complexity of ACP in this population and act as barriers for clinicians and carers, namely, fluctuating capacity and persistent symptoms of mental illness. As such, there was a focus in the handouts on assessing capacity alongside strategies to empower the consumer, such as supported decision making. To facilitate access, these written resources were made freely available on the Capacity Australia website. (https://capacityaustralia.org.au/acp-resources/).

Common elements for each stakeholder information sheet included information about what ACP is, why it is important, when it should be considered, dispelling misconceptions (specific to those raised by that group in the primary analysis) and resources for further information. The consumer version contained additional information on whom to seek help from with ACP and practical steps in ACP. Carer and clinician versions included information on decision-making capacity and the law. Specific tips on how to help a consumer with ACP from the perspective of a Carer or Clinician were included in respective versions. To address carer-identified concerns about ACP and knowledge gaps highlighted in the qualitative analysis, targeted topics addressing these issues were provided in the carer information sheet (https://capacityaustralia.org.au/wp-content/uploads/2025/03/MT0328_NSWGOV_SLHD01_BROCHURE_V6_%E2%95%9EAE_CARER_WEB.pdf),

Culture, ethnicity and religion emerged as pertinent to ACP in the thematic analysis (Wand et al. 2024, 2025). Recognizing that not all consumers and carers are literate in English, the ACP information sheets were translated into the three most common languages spoken in Sydney Local Health District; Simplified Chinese, Arabic and Greek (https://capacityaustralia.org.au/acp-resources/). Translation was undertaken by the NSW Multicultural Health Communication Service.

The stakeholder information sheets were presented to mental health clinicians for feedback, who rated the information sheets as having good face validity. Modifications were suggested and incorporated regarding the content, font size, layout and information to emphasize for consumers, to improve relevance, readability and focus on key messages.

- Skill gaps: how to do it

*“If you don’t do it very often, then you’re not very skilled at it. And so, then it comes out as very awkward when you do try to do it and that makes you not really wanna try again.”* (psychiatrist) (Wand et al. 2024)

*“The professors, doctors, social workers … you wouldn’t [need special skills] in your positions, you’d have that skill already.”* (male consumer, aged 69) (Wand et al. 2025)

Consumers and carers thought that mental health clinicians had the requisite skills and personal qualities to assist consumers with ACP, so would not need any special training. This conflicted with a theme emerging from analysis of clinician focus groups, that they lacked knowledge and practical training in ACP. This skill gap was also reflected by their concerns about causing consumers harm (e.g., by inadvertently causing offence or exacerbating psychotic symptoms) by raising ACP, especially from not knowing how or when to do it, and difficulty navigating legal issues and capacity, particularly with consumers who have residual symptoms of mental illness.

### Matched intervention

As a key gap identified was practical skills engaging in ACP with consumers, training films were developed which illustrated common misconceptions and perceived challenges and demonstrated strategies to address them. The scripts were co-written by an occupational therapist with extensive practical experience of ACP with people with mental illness and/or dementia and two psychogeriatricians. The content was drawn directly from the original qualitative research (Wand et al. 2024, 2025), emphasizing practical challenges of implementing ACP with this specific cohort (e.g. persistent psychotic symptoms, timing of ACP discussions in relation to acuity of mental illness) and techniques to address them. Accompanying the scripts were “pop up boxes” of key learning points overlaid on the film. Film length was capped at 10 minutes each to be easily digestible for a busy clinician.

A design and production company, Bright Agency, with expertise in communication and health promotion was hired to create the films. Volunteers were recruited to play the roles of consumers and carer. A senior clinician (RB) with clinical experience in ACP played the role of mental health clinician in both films.

Two films were created. Each film demonstrates how a clinician may introduce, explain and encourage ACP with a consumer. One film illustrates how to engage and adapt ACP for an older adult consumer with stable residual psychotic symptoms and cognitive impairment (https://capacityaustralia.org.au/acp-resources/). The other film depicts ACP with a consumer recovering from psychotic depression and his wife, highlighting how to determine the timing of ACP in relation to illness course, approaches to addressing carer concerns about causing harm, and dispelling misconceptions (https://capacityaustralia.org.au/acp-resources/).

The 2 films were played to a convenience sample of 13 people including older adults and carers of older adults, general community members and multidisciplinary clinicians. Spontaneous discussion of the importance of ACP followed the screening. Feedback indicated that the films appeared authentic (reflective of real-world clinical interactions), were engaging, well produced and clearly conveyed key messages. Some perceived the film lengths as too long.

To complement the practical demonstration of ACP in the films, suggestions for starting ACP discussions were also included in the clinician information sheet, alongside information on assessing capacity and determining the right time for ACP discussions in the context of a person’s acuity of mental illness (https://capacityaustralia.org.au/wp-content/uploads/2025/03/MT0328_NSWGOV_SLHD01_BROCHURE_V6_%E2%95%9EAE_CLINICIAN_WEB.pdf).

- Practical and process issues

*“…because I’ve got nobody else to discuss this with…. So [it would be] good to talk to someone about advance care planning.… to have a conversation with a health professional.”* (male consumer, 68) (Wand et al. 2025)

*“The attitude is really important, the enthusiasm. [The mental health clinician] is so close, friendly, and spends time with [the consumer]. They care….”* (son) (Wand et al. 2025)

*“Who’s got the time?”* (social worker) (Wand et al. 2024)

*“I don’t know if there’s a policy. I don’t know what the standard practice is.”* (occupational therapist) (Wand et al. 2024)

Practical and process aspects of engaging consumers in ACP within usual clinical care emerged as important themes across stakeholder groups. Flexibility with ACP was identified by consumers and carers as important, including their having a say in where, when and how ACP takes place. Difficulty determining the optimum timing of ACP discussions was perceived as a barrier to clinicians engaging in ACP.

All three stakeholder groups identified time as an obstacle to engaging in ACP. Consumers and carers perceived General Practitioners (GPs) as too busy to discuss ACP. Related to this theme was divergence among stakeholder groups as to who should lead ACP with consumers. Consumers and carers identified mental health clinicians as inherently having the skills, knowledge of and therapeutic relationship with the consumer and time for ACP. By contrast, clinicians were unsure ACP was their role, did not see it as a priority and described lacking the time required to engage in ACP.

An emergent theme from families/carers was their potential role as cultural brokers and leveraging their in-depth knowledge of the consumer (e.g., their mental health or trauma history) to support ACP. This role provides a potential solution to the complexity of considering culture identified by clinicians and contrasts with the clinician concerns about families and culture as potential barriers to ACP. Carer involvement in ACP may also mitigate their identified fears of clinicians inadvertently causing harm if they lacked sensitivity or did not carefully consider the right time for ACP discussions with consumers.

Clinicians raised the need for systemic change to support their involvement in ACP. The theme of ACP not being prioritized or embedded in practice was a barrier as clinicians were unsure of their roles and responsibilities in relation to ACP. Clinicians observed deficiencies in procedural guidance and access to local policies on ACP which amplified their uncertainty about implementation. Lack of knowledge about where and how to document ACP discussions were raised.

Consumers wanted their ACP discussions shared widely, including with their GP, support services and families. Carers echoed this sentiment. This did not emerge from analysis of clinician data, perhaps due to uncertainties about their role in ACP and documentation of discussions.

### Matched intervention

Proposed strategies to optimize the timing of ACP discussions informed by the original qualitative studies included leveraging the combined expertise of the multidisciplinary team available to mental health clinicians (person-centered risk analysis of timing), consulting the treating psychiatrist about questions of capacity, and incorporating carer/family knowledge of the consumer (Wand et al. 2024, 2025). These strategies were included in the stakeholder information sheets (carers and clinicians). The film featuring the couple and clinician discussing ACP highlighted ways of providing initial information on ACP to evaluate whether this is the right time for ACP, ultimately determining more time was needed for recovery from depression before the consumer was able to comfortably and meaningfully participate in ACP, and respecting the wife’s input that now was not the right time.

Solutions were proposed to address the issue of lack of time for ACP. A consumer participant proposed having a series of short discussions about ACP with a clinician rather than one long session. This aligned with a solution proposed from the analysis of clinician data, to have iterative brief discussions with consumers over a series of sessions (Wand et al. 2024), recommended elsewhere (Knippenberg et al. 2023). This strategy of “evolving conversations” is included in the information sheet for clinicians. It is also reflected in the training films, which both demonstrate brief conversations about ACP contained within 10 minutes and plans to revisit discussions later.

Cultural aspects were identified as relevant to ACP, both as an aspect of complexity and barrier to ACP but also a factor which might promote engagement in ACP if incorporated sensitively. All stakeholder information sheets include information on who can help and support consumers in ACP. The carer information sheet notes the potential role of the carer supporting ACP through their knowledge about the consumer’s personal history, cultural background and care needs. Both films illustrate the utility of involving nominated carers/family in ACP.

Clinicians identified a need for systemic change if ACP is to be more broadly incorporated into clinical practice. Accordingly, the clinician information sheet has a dedicated section on how ACP may be brought into routine care, including through being embedded in care plans and ward rounds or community multidisciplinary team discussions. The information sheets may also be used to support implementation, as a simple stakeholder-informed resource which can be given by clinicians to consumers and carers to start or follow-up ACP discussions. Other strategies to effect systemic change included support from managers emphasizing that ACP is important and part of routine care, backed-up by guidelines and procedures to support implementation (Wand et al. 2024). Appointing team-based ACP champions and using audit and feedback would help reinforce ACP as part of standard care (Wand et al. 2024). The need for accessible documentation of ACP (e.g., in the medical records) is also highlighted, alongside sharing ACP information widely, the latter consistent with the expressed wishes of consumers (Wand et al. 2025) and improving the likelihood of ACP information being identified and utilized when needed towards the end of life.

## Discussion

To the best of our knowledge this is the first study to describe the development of evidence-informed educational resources to support older adults with mental illness, their carers and clinicians with ACP. Four overarching themes emerged in triangulation of qualitative analyses from each of the three stakeholder groups (Wand et al. 2024, 2025): (i) importance of ACP recognized but ACP often not initiated; (ii) knowledge gaps; (iii) skill gaps – how to do it; and (iv) practical and process issues. Educational resources were developed to address the identified issues within each overarching theme; information sheets bespoke to each stakeholder group and two clinician training films demonstrating key aspects of ACP in practice. The resources were designed to bridge specific knowledge and skill gaps, but also to be practical providing guidance for implementation of ACP into mental health clinical practice, supporting broader educational interventions and complemented by governance strategies to embed ACP within health services.

The literature describing educational resources for mental health clinicians in ACP while limited, is promising. The “Do It Your Way” project demonstrated the ability and interest of mental health consumers to engage in ACP, without causing harm (Foti et al. 2005), and was associated with an increase in completion of ACP documents and health care proxies (Foti 2003). However, the resources developed for the project are not freely available and do not appear to have been derived empirically precluding comparison with our resources (Foti 2003). A 3-hour 1-time workshop for psychiatry residents on medical and psychiatric ACP was described in abstract form only (Bates et al. 2019). The workshop included drill-based practice of ACP communication skills and role-playing completion of Advance Care Directives (ACD) using cases. For the 41 participants who completed post-workshop surveys, objective measures of ACP knowledge improved, alongside self-reported greater comfort discussing end-of-life care, ability to address key elements of ACP, and facilitating medical ACP – all statistically significant (Bates et al. 2019). No details were provided on whether there were take-home/online resources to reinforce the teaching. An Australian study described cross-training mental health and palliative care clinicians through liaison roles, education and modelling, with ACP among the workshop topics for 65 mental health workers (Taylor et al. 2012). Knowledge gains were reported, although not quantified or specific to ACP.

We were unable to find any studies reporting the development of and sharing plain-language resources to educate mental health consumers and carers about ACP. Although there are overlapping education and training needs to support ACP in mental health (Jerwood et al. 2018; Wand et al. 2024, 2025) and general population contexts (Poveda-Moral et al. 2021), there are particular considerations and challenges to implementation which emerge with serious mental illness necessitating population-specific resources and training for mental health clinicians.

## Limitations

Although clinician feedback was sought regarding the stakeholder information sheets and incorporated into the final versions, consumers and carers were not asked to formally evaluate these documents. This was largely due to time pressure completing the study but is important to ensure that the resources are understandable and useful for consumers and carers. Similarly, the training films were not evaluated by consumers, although they were primarily designed to assist clinicians in practical aspects of engaging consumers in ACP.

The content of the education resources was informed by interviews with consumers with a psychotic illness and their carers and so may not be generalizable to older adults with other mental illnesses. Although arguably the identified complexity conferred by psychotic symptoms in relation to consumer education, clinician concerns about causing distress and the challenges of evaluation of capacity in ACP, are relevant to mental health consumers more broadly (McNamara et al. 2018; Wand et al. 2025). While our resources are available in four languages, funding constraints limited expansion to other languages. Access to appropriate resources is only one factor which may support ACP in culturally and linguistically diverse communities. We did not explore specific cultural factors impacting ACP, care settings and physical environments and particular skills needed in clinicians and interpreters; all noted to be important in implementation of ACP with ethnically diverse people with cancer (Chauhan et al. 2025). Finally, use of these new evidence-informed resources has not yet been formally tested within clinician training on ACP. However, a knowledge translation study is underway comprising interactive education sessions (combining didactic information with application to clinical practice), supported by our training resources and strategies for organizational change.

Solving the problem of ACP implementation with people with mental illness is complex. This study used thematic analysis of stakeholder voices to elucidate the barriers to ACP which were then used to determine what is needed to address the underlying issues, namely, education and training. The resources address some barriers to engagement in ACP and are designed as supporting tools to be used in combination with other strategies such as organizational change (addressing the theme of practical and process issues) and a comprehensive dedicated educational intervention. Our participants did not highlight other potentially useful strategies such as public health campaigns (Seymour 2018), policy work (for example, https://www.health.nsw.gov.au/patients/acp/Publications/final-report-acp-action-plan.pdf), financial support or training offensives targeting clinicians, which warrant exploration.

In conclusion, advance care planning is uncommon in older people with mental illness despite high rates of physical morbidity and premature mortality (Firth et al. 2019). Previous qualitative research with older adult consumers and their carers highlighted their expectation ACP would be raised with them if important (Wand et al. 2025) – but it is not. Mental health clinicians have therapeutic relationships and the trust of consumers which may facilitate ACP (Wand et al. 2025) and the opportunity to facilitate links to general medical care (Foti et al. 2005), but do not currently engage in ACP for complex reasons (Wand et al. 2024). Key strengths of this study were turning these empirically derived stakeholder barriers into specific actions to facilitate ACP, by creating resources bespoke to different stakeholder needs to support implementation at practical and governance levels in public mental health settings. The resources are one component of what will need to be a multipronged strategy to improve engagement in ACP in this population. To have an impact, dedicated educational interventions for clinicians must be accompanied by organizational change, such as embedded processes to support, maintain and monitor implementation and leadership support (Wand et al. 2024), identified as core components of effective educational interventions in other aspects of healthcare (Lee et al. 2020). Improving consumer and carer awareness and knowledge about ACP should also be a focus (Chen et al. 2025; Wand et al. 2025), promoting empowerment and advocacy respectively.

The next step is to evaluate the effectiveness of these resources in clinical practice with mental health consumers and carers as part of a more comprehensive educational intervention training clinicians in ACP. Improving rates of ACP in this cohort is an achievable and measurable outcome, addressing a core human right of equitable access to self-determination and facilitation of quality care towards the end of life (Peisah et al. 2021).
